# Response of *Salmonella enterica* serovar Typhimurium to alginate oligosaccharides fermented with fecal inoculum: integrated transcriptomic and metabolomic analyses

**DOI:** 10.1007/s42995-023-00176-z

**Published:** 2023-05-29

**Authors:** Jiaying Cheng, Mengshi Xiao, Xinmiao Ren, Francesco Secundo, Ying Yu, Shihao Nan, Weimiao Chen, Changliang Zhu, Qing Kong, Youtao Huang, Xiaodan Fu, Haijin Mou

**Affiliations:** 1 College of Food Science and Engineering, Ocean University of China, Qingdao, 266003 China; 2 Istituto di Scienze e Tecnologie Chimiche “Giulio Natta”, Consiglio Nazionale delle Ricerche, 20131 Milan, Italy; 3 State Key Laboratory of Food Science and Technology, China-Canada Joint Laboratory of Food Science and Technology (Nanchang), Key Laboratory of Bioactive Polysaccharides of Jiangxi Province, Nanchang University, Nanchang, 330047 China; 4 School of Medicine and Pharmacy, Ocean University of China, Qingdao, 266003 China

**Keywords:** Alginate oligosaccharides, Gut microbiota, Metabolite, *Salmonella enterica* serovar Typhimurium, Metabolomics, Transcriptomic

## Abstract

Alginate oligosaccharides (AOS), extracted from marine brown algae, are a common functional feed additive; however, it remains unclear whether they modulate the gut microbiota and microbial metabolites. The response of *Salmonella enterica* serovar Typhimurium, a common poultry pathogen, to AOS fermented with chicken fecal inocula was investigated using metabolomic and transcriptomic analyses. Single-strain cultivation tests showed that AOS did not directly inhibit the growth of *S*. Typhimurium. However, when AOS were fermented by chicken fecal microbiota, the supernatant of fermented AOS (F-AOS) exhibited remarkable antibacterial activity against *S*. Typhimurium, decreasing the abundance ratio of *S*. Typhimurium in the fecal microbiota from 18.94 to 2.94%. Transcriptomic analyses showed that the 855 differentially expressed genes induced by F-AOS were mainly enriched in porphyrin and chlorophyll metabolism, oxidative phosphorylation, and *Salmonella* infection-related pathways. RT-qPCR confirmed that F-AOS downregulated key genes involved in flagellar assembly and the type III secretory system of *S*. Typhimurium, indicating metabolites in F-AOS can influence the growth and metabolism of *S*. Typhimurium. Metabolomic analyses showed that 205 microbial metabolites were significantly altered in F-AOS. Among them, the increase in indolelactic acid and 3-indolepropionic acid levels were further confirmed using HPLC. This study provides a new perspective for the application of AOS as a feed additive against pathogenic intestinal bacteria.

**Supplementary Information:**

The online version contains supplementary material available at 10.1007/s42995-023-00176-z.

## Introduction

Antibiotics are used in the animal breeding industry to improve growth performance and animal health (Tasho and Cho 2016); however, long-term and uncontrolled use of antibiotics has led to the accumulation of residual antibiotics, antibiotic resistance in pathogens, and an imbalance in the normal microbiota of poultry (Cheng et al. 2014). Several alternatives have been proposed to overcome the adverse effects of the indiscriminate use of antibiotics in livestock breeding and aquaculture, including the application of antimicrobial peptides, functional oligosaccharides and dietary fibers (Nawaz et al. 2018; Li et al. 2020a, b). Recently, oligosaccharides have attracted attention as promising alternatives to antibiotics in the breeding industry (Lindberg 2014). Non-digestible oligosaccharides (NDOs) are intact when they reach the colon and are utilized by specific microbial groups (Ramakrishna 2013). Previous studies have reported the ability of oligosaccharides to increase the number of short-chain fatty acids (SCFAs) in the intestinal tract, inhibit pathogen adhesion, and repair the intestinal mucosa (Ghasemian and Jahanian 2016; Macfarlane et al. 2008; Yan and Ganzle 2018).

The most commonly reported pathogenic or zoonotic bacteria in poultry include *Salmonella enterica*, *Escherichia coli*, and *Clostridium perfringens*, which can colonize the intestine and negatively affect animal health, growth, and reproductive performance (Ng and Koh 2017). Therefore, therapies targeting the intestinal environment can decrease the risk of disease, thereby improving the ability of poultry to prevent pathogens from colonizing the intestine, adhering to the intestinal mucosa, and releasing toxins. The main site of *Salmonella* infection in poultry is the intestinal tract, which leads to the contamination of carcasses and eggshells. Specifically, *Salmonella enterica* serovar Typhimurium (*S*. Typhimurium), one of the most common enteric pathogenic bacteria, causes serious economic losses in the poultry sector and is associated with foodborne outbreaks owing to human consumption of contaminated poultry products (Lv et al. 2021). Thus, preventive treatment of *S*. Typhimurium infection and colonization is essential for blocking pathogenic contamination in poultry (Evans et al. 2017).

Alginate is a linear and acidic polysaccharide commonly isolated from brown algae. Alginate oligosaccharides (AOS), composed of β-d-mannuronic acid (M) and α-l-guluronic acid (G) units linked by β-1,4-glycosidic bonds, are enzymatically degraded or hydrolyzed from alginate (Cao et al. 2021). Moreover, AOS exhibit biological properties, such as anti-inflammatory, immunoregulatory, and antibacterial properties (Xing et al. 2020). AOS disrupted *Pseudomonas aeruginosa* biofilm assembly, which might primarily result from its ability to reduce the synthesis of acyl-homoserine lactone (Jack et al. 2018). Notably, AOS decreased the pathogenicity of *Candida albicans* by suppressing the hyphal formation and reducing phospholipase activity (Pritchard et al. 2017). The use of AOS as a feed additive for poultry and livestock was also investigated. AOS modulated the gut microbiota, increased the production of SCFAs, and decreased the levels of endotoxins, thus playing an important role as an antibacterial agent in poultry breeding (Wang et al. 2020). For example, Zhu et al. (2015) assessed the effects of AOS on the cecal fermentation profile of broilers and showed that AOS increased the abundance of lactic acid bacteria, reduced the abundance of *E*. *coli*, and increased the SCFA (e.g., acetic acid and butyric acid) and lactic acid contents. As an animal dietary additive, AOS also increased the relative abundance of *Bifidobacterium*, *Coprococcus*, and *Butyricicoccus* (Han et al. 2022). However, the complex mechanisms involved in the metabolic changes caused by AOS and the mechanisms underlying its antibacterial activity in the intestine remain unclear. Research on AOS as a direct inhibitor of pathogenic bacteria is lacking. Therefore, elucidating how AOS affected the gut metabolism in poultry would assist in the development of new treatments to overcome the adverse effects of excessive antibiotic use.

In our previous study, AOS (Mw: 1.93 kDa, M/G ratio = 0.37) were prepared by enzymatic degradation (Yang et al. 2018). In the present study, the profiles of AOS fermented with chicken fecal microbiota in vitro and the antibacterial mechanism of AOS against *S*. Typhimurium were investigated. Moreover, the antibacterial effects of AOS fermentation supernatant with chicken fecal inoculum (F-AOS) were evaluated. Key genes related to growth and virulence factor expression in *S*. Typhimurium treated with F-AOS were evaluated using transcriptomic analysis, and untargeted metabolomics was used to elucidate the effects of AOS on gut metabolites. This study provides a theoretical basis for the use of AOS as an antibiotic substitute in livestock.

## Results

### AOS fermentation by chicken fecal microbiota in vitro

In this study, the utilization of AOS was compared with that of galacto-oligosaccharide (GOS) and glucose. AOS were utilized and fermented by chicken fecal microbiota; however, bacterial growth and acid production were weaker than those in the glucose and GOS groups (Supplementary Fig. S1A). GOS and glucose are carbohydrates that are rapidly utilized by the gut microbiota. The addition of glucose promoted rapid bacterial growth, with the OD_600_ increasing from 0.072 at 0 h to 0.380 at 24 h. At 48 h, the GOS group showed obvious growth, whereas AOS treatment did not promote bacterial growth until after 120 h of fermentation. The growth of bacteria (OD_600_) reached a peak at 96 h after treatment with GOS (0.405) and glucose (0.644) then began to decline, indicating the carbon source was rapidly consumed. In addition, both glucose and GOS exhibited a high rate of acid production, with the pH decreasing to 3.5 and 3.7 at 48 h, respectively (Supplementary Fig. S1B), whereas the AOS group showed a non-significant decrease in pH. Thin-layer chromatography (TLC) was used to determine the oligosaccharide-utilization patterns during fermentation (Supplementary Fig. S1C-D). The results confirmed that AOS could be fermented and selectively utilized by the chicken fecal microbiota. The dots representing trisaccharides (*III*), tetrasaccharides (*IV*), pentasaccharides (*V*), and hexasaccharides (*VI*) in the chromatogram were almost absent after 144 h of incubation, indicating that these components in AOS were completely utilized.

### Detection of the antibacterial activity of AOS against *S*. Typhimurium in chicken fecal cultures

**Fig. 1:**
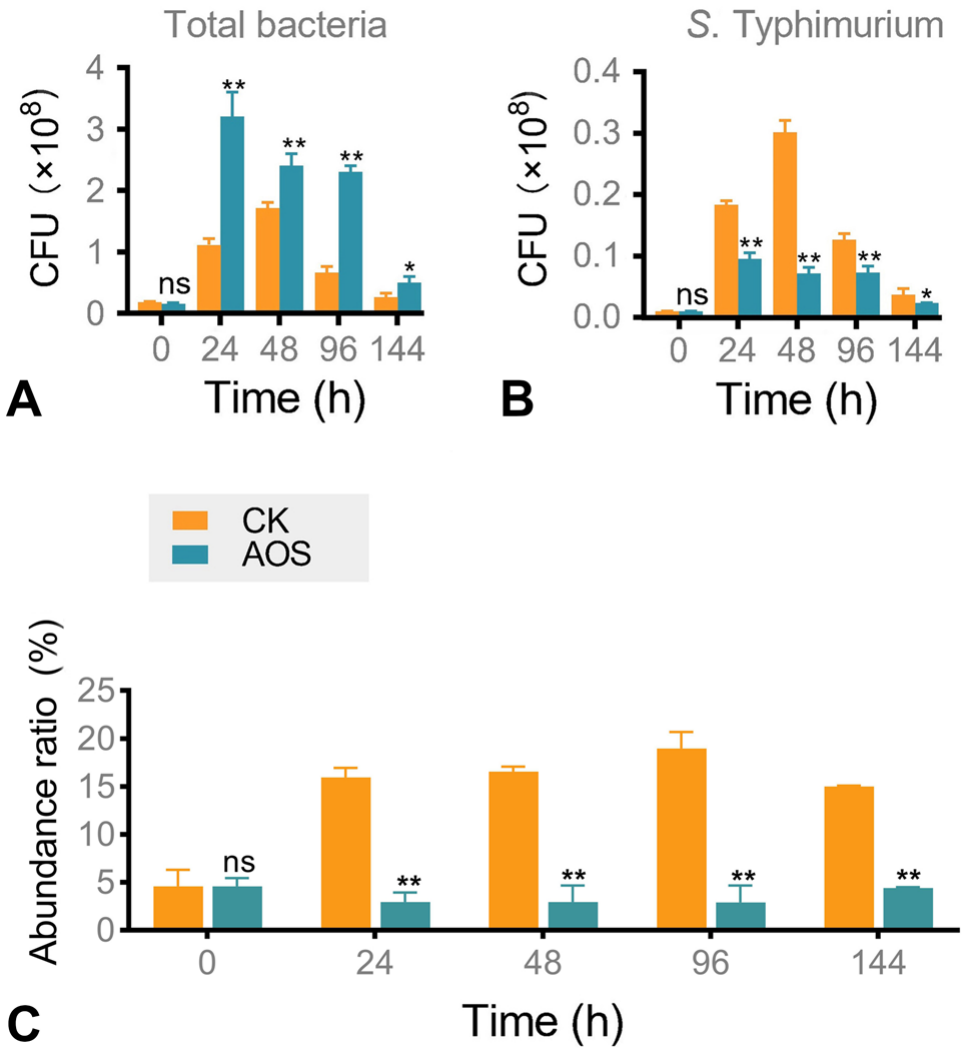
Inhibitory effect of AOS against *S*. Typhimurium during in vitro chicken fecal fermentation. **A** Total bacterial colony count of gut microbiota. **B** *S*. Typhimurium colony count in gut microbiota. **C** The abundance ratios of *S*. Typhimurium in the chicken gut microbiota. CK, without adding any oligosaccharides. Statistical significance was determined by Student’s *t*-test (*n* = 3). ns, nonsignificant; *, *P* < 0.05; **, *P* < 0.01

To detect the antibacterial activity of AOS against *S*. Typhimurium in chicken fecal cultures, AOS were in vitro fermented by chicken fecal inocula supplemented with *S*. Typhimurium. Rapid growth of *S*. Typhimurium was observed in the control group. The abundance ratio of *S.* Typhimurium increased from 4.57% at 0 h to 15.94% at 24 h in the control group, which was significantly higher than that in the AOS-treated group (2.92%; *P* < 0.01). The abundance ratio of *S*. Typhimurium in the fecal microbiota decreased from 18.94% to 2.94% in the AOS-treated group at 96 h, showing the most significant antibacterial effect (Fig. 1). This indicated AOS inhibited the growth of *S*. Typhimurium, limiting the increase in *S*. Typhimurium ratio in the chicken gut microbiota. Whether AOS directly inhibits *S*. Typhimurium or plays a bacteriostatic role through the metabolism of intestinal microbiota will be the focus of follow-up work.

### Antibacterial mechanism of AOS against *S*. Typhimurium

**Fig. 2:**
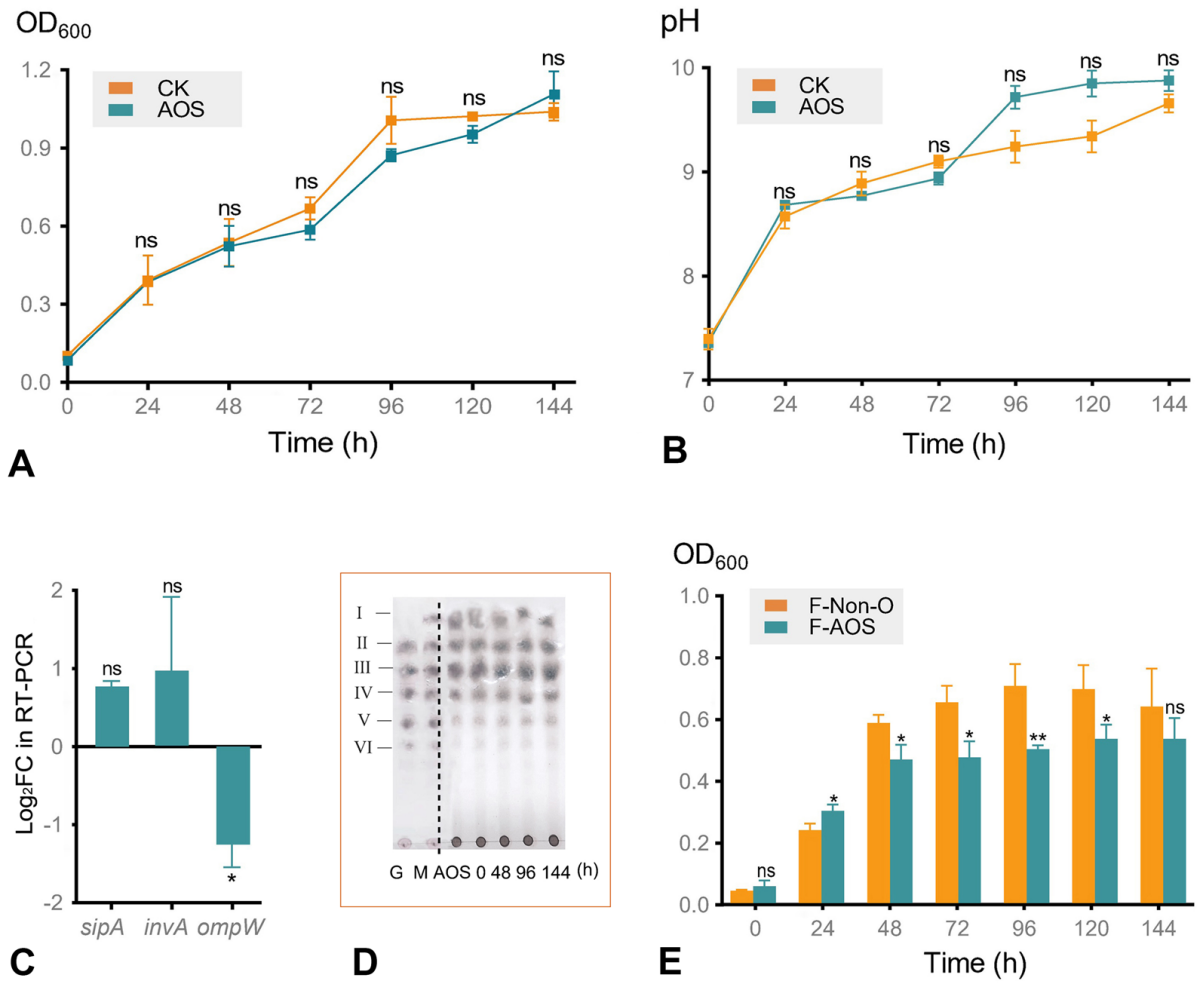
Antibacterial activity of AOS and F-AOS during single *S*. Typhimurium cultivation. A-D,* S*. Typhimurium was treated with AOS. E, *S*. Typhimurium was treated with F-AOS. **A** Growth curves of *S*. Typhimurium. **B** Changes of pH values. **C** The mRNA level of pathogenicity related genes (*sipA*, *invA*, and *ompW*) were assessed using RT-qPCR. **D** TLC profiles of the utilization of AOS at 0, 48, 96, and 144 h, respectively. **E** OD_600_ of *S*. Typhimurium in F-AOS and F-Non-O. CK, control group; M, mannuronate oligosaccharides (DP 1–6); G, guluronate oligosaccharides (DP 2–6); *I*-*VI*, DP 1–6; F-AOS, supernatant of AOS fermented with chicken fecal inoculum. F-Non-O, supernatant of fermented non-O. Significant differences among substrates at the same time point were calculated using Student’s *t*-test (*n* = 3). ns, nonsignificant; *, *P* < 0.05; **, *P* < 0.01

These experiments demonstrated that the addition of AOS to the intestinal microbiota inhibited the proliferation of *S*. Typhimurium. Can AOS directly inhibit the growth and metabolism of *S*. Typhimurium? To elucidate the mechanism of this antibacterial effect, the growth of *S*. Typhimurium during single-strain cultivation was assessed. The growth profile of *S*. Typhimurium in the presence of AOS is shown in Fig. 2A. After 144 h of fermentation, all groups showed significantly increased OD_600_ (1.040–1.100), with no significant differences between the AOS and the control groups. The changes in pH were also not significantly different between the two groups (Fig. 2B). To further illustrate the response mechanism of *S*. Typhimurium against AOS, representative pathogenicity-related genes of *S*. Typhimurium, including *sipA*, *invA*, and *ompW*, were selected for reverse transcription quantitative PCR (RT-qPCR) analysis (Fig. 2C, Supplementary Table S1). The expression levels of *sipA* and *invA* were not significantly different between the two groups. However, mRNA levels of *ompW* were significantly reduced by AOS treatment (*P* < 0.05). TLC was also used to verify the utilization of AOS by *S*. Typhimurium, and the dots representing AOS remained almost unchanged during 144 h of fermentation (Fig. 2D). The evidences showed that AOS did not directly inhibit the growth of *S.* Typhimurium and could not be utilized during single *S*. Typhimurium cultivation.

### Antibacterial effect of fermented AOS supernatant against *S*. Typhimurium during single-strain cultivation

Based on the non-significant inhibitory effect of AOS against *S*. Typhimurium during single-strain cultivation, we hypothesized that AOS metabolites exert an inhibitory effect on *S*. Typhimurium during fecal fermentation. Therefore, the antibacterial activity of F-AOS against *S*. Typhimurium during single-strain cultivation was elucidated (Fig. 2E). Medium without any oligosaccharides was fermented by fecal microbiota (non-O), and the supernatant was used as the control (F-Non-O). F-AOS exhibited a significantly lower OD_600_ (0.450) than F-Non-O at 48 h (0.590; *P* < 0.05). The maximum antibacterial effect appeared at 96 h, at which point the OD_600_ value of *S*. Typhimurium in the control group was 1.41-fold that in the F-AOS group. The AOS fermentation metabolites showed sustained antibacterial activity throughout the experimental period.

### Transcriptomic analysis to elucidate the response of* S*. Typhimurium against F-AOS

**Fig. 3:**
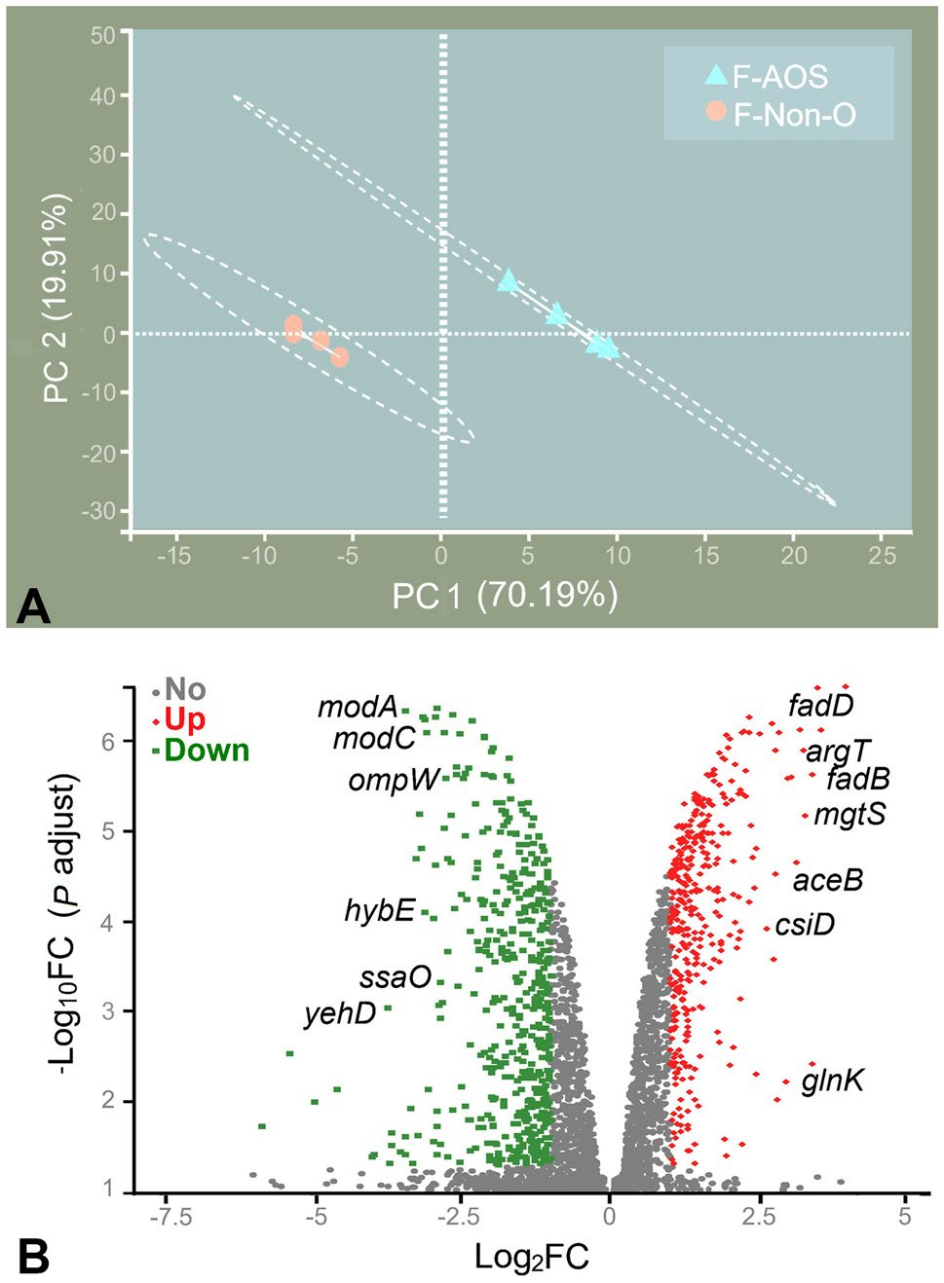
Effects of F-AOS on *S*. Typhimurium transcriptomics. **A** PCA plot. **B** Volcano graph of 855 DEGs. Red represents upregulated DEGs; green represents downregulated DEGs. F-AOS, supernatant of AOS fermented with chicken fecal inoculum. Supernatant of non-O fermented (F-Non-O) was used as the control

To evaluate the inhibitory mechanism of AOS metabolites fermented with chicken fecal inoculum, the response of *S*. Typhimurium to F-AOS was analyzed at the mRNA level using transcriptomic analysis.* S*. Typhimurium treated with F-AOS and F-Non-O were subjected to whole transcriptome sequencing (RNA-seq). A total of 22.95–29.34 million clean reads were obtained from each sample after the quality check (Supplementary Table S2). Principal component analysis (PCA) plots based on normalized read counts showed a clear separation between the transcriptomic profiles of *S*. Typhimurium in F-AOS and F-Non-O groups (Fig. 3A). PC1 and PC2 filtering separated the two groups, accounting for 70.19% and 19.91% of the total variation, respectively. Differential gene expression analysis was performed to identify the differentially expressed genes (DEGs). A total of 855 DEGs were obtained in the F-AOS group compared with their expression in the F-Non-O group, with filter criteria being fold-change (FC) > 2 or < -2 and *q* value < 0.05; 370 DEGs were upregulated and 485 DEGs were downregulated (Fig. 3B, Supplementary Table S3).

**Fig. 4:**
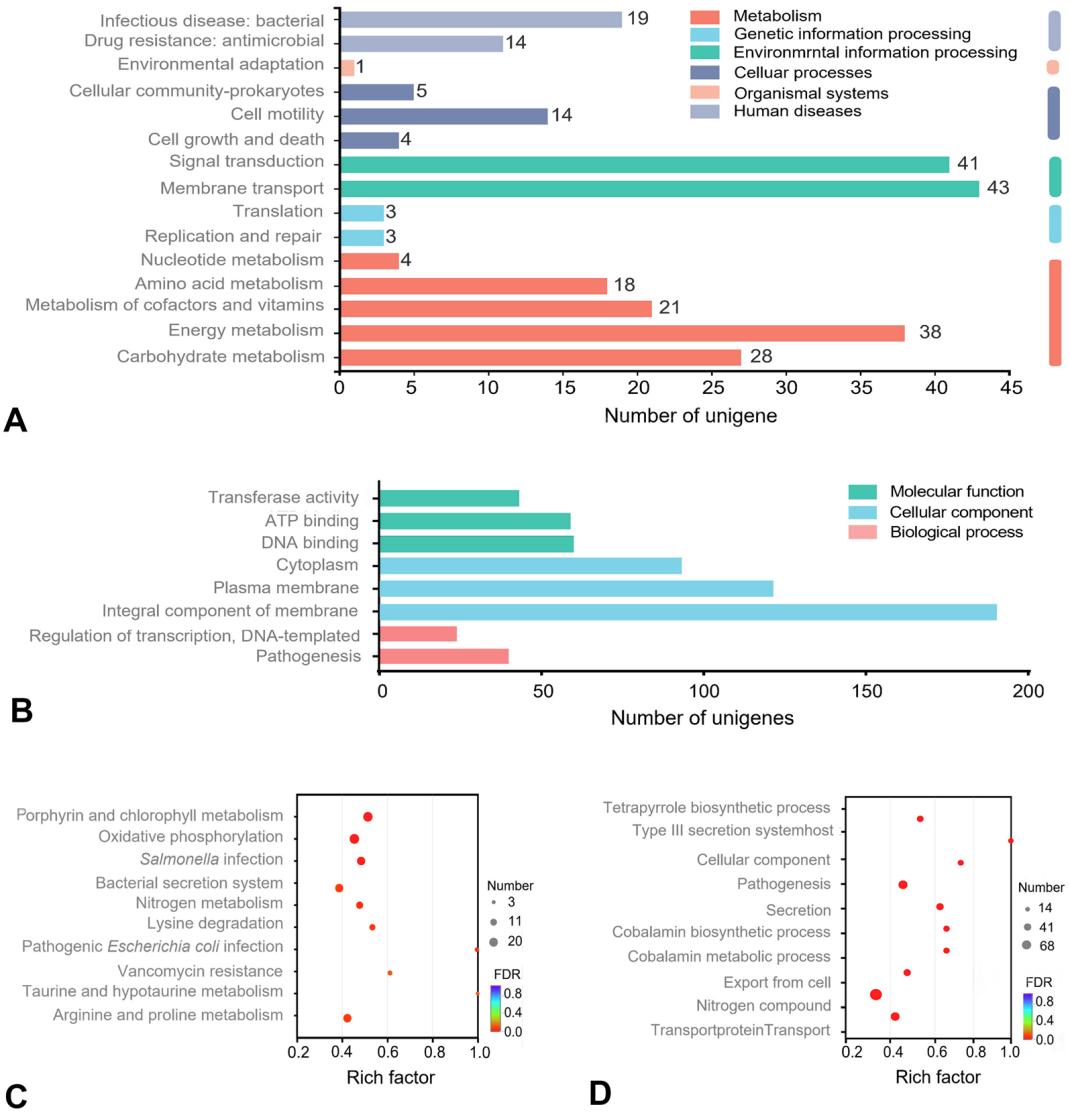
KEGG and GO analysis. **A** KEGG pathway classification of genes. **B** GO classifications of genes. **C** KEGG pathway enrichment analysis. **D** GO enrichment analysis. Rich factor, number of differentially expressed genes total number. F-AOS, supernatant of AOS fermented with chicken fecal inoculum. Supernatant of non-O fermented (F-Non-O) was used as the control

The DEGs were successfully annotated to 131 pathways. Kyoto Encyclopedia of Genes and Genomes (KEGG) analysis revealed that 43, 41, 38, 28, and 21 DEGs were mainly involved in membrane transport, signal transduction, energy metabolism, carbohydrate metabolism, and metabolism of cofactors and vitamins, respectively (Fig. 4A). The genes annotated in the “membrane transport” pathway that were significantly downregulated belonged to the *ssa* family (*ssaU*, *ssaT*, *ssaA*, *ssaR*, *ssaQ*, *ssaJ*, and *ssaG*) and *mod* family (*modA*, *modB*, and *modC*), being related to type III secretion system and ABC transporter, respectively. Gene ontology (GO) database annotation revealed that the eight most significantly enriched terms included molecular functions (three subcategories), cellular components (three subcategories), and biological processes (two subcategories). The molecular functions mainly focused on ATP binding, DNA binding, and transferase activity. Significant cellular components from GO terms were included as integral components of the membrane, plasma membrane, and cytoplasm. Significant changes in biological processes included pathogenesis and regulation of transcription and DNA templates (Fig. 4B). Regarding the “integral components of the membrane” pathway, F-AOS treatment caused significant upregulation of 38 genes and downregulation of 152 genes. Among them, *modB* and *ompW*, encoding the molybdate ABC transporter permease subunit and outer membrane protein OmpW, respectively, showed the most downregulated levels (log_2_ FC -3.17 and -3.07, respectively); *mgtS* and *fadE*, encoding protein MgtS and acyl-CoA dehydrogenase FadE, respectively, showed the most upregulated levels (log_2_ FC 3.37 and 2.23). Pathway enrichment analysis further elucidated the biological functions of the DEGs; four KEGG pathways were significantly enriched (*P* < 0.05; Fig. 4C). A relatively large number of DEGs were involved in porphyrin and chlorophyll metabolism (map00860), oxidative phosphorylation (map00190), *Salmonella* infection (map05132), and pathogenic *Escherichia coli* infection (map05130). In the “*Salmonella* infection” pathway, 15 genes were significantly downregulated, all of which were concentrated in the *Salmonella* pathogenicity islands. GO enrichment analysis was performed to characterize significant functional DEGs induced by F-AOS treatment (Fig. 4D). The tetrapyrrole biosynthetic process (GO: 0033014), protein secretion by the type III secretion system (GO: 0030254), host cellular components (GO: 0018995), and pathogenesis (GO: 0009405) were the enriched “biological processes” terms.

### Untargeted metabolomic profile of F-AOS

**Fig. 5:**
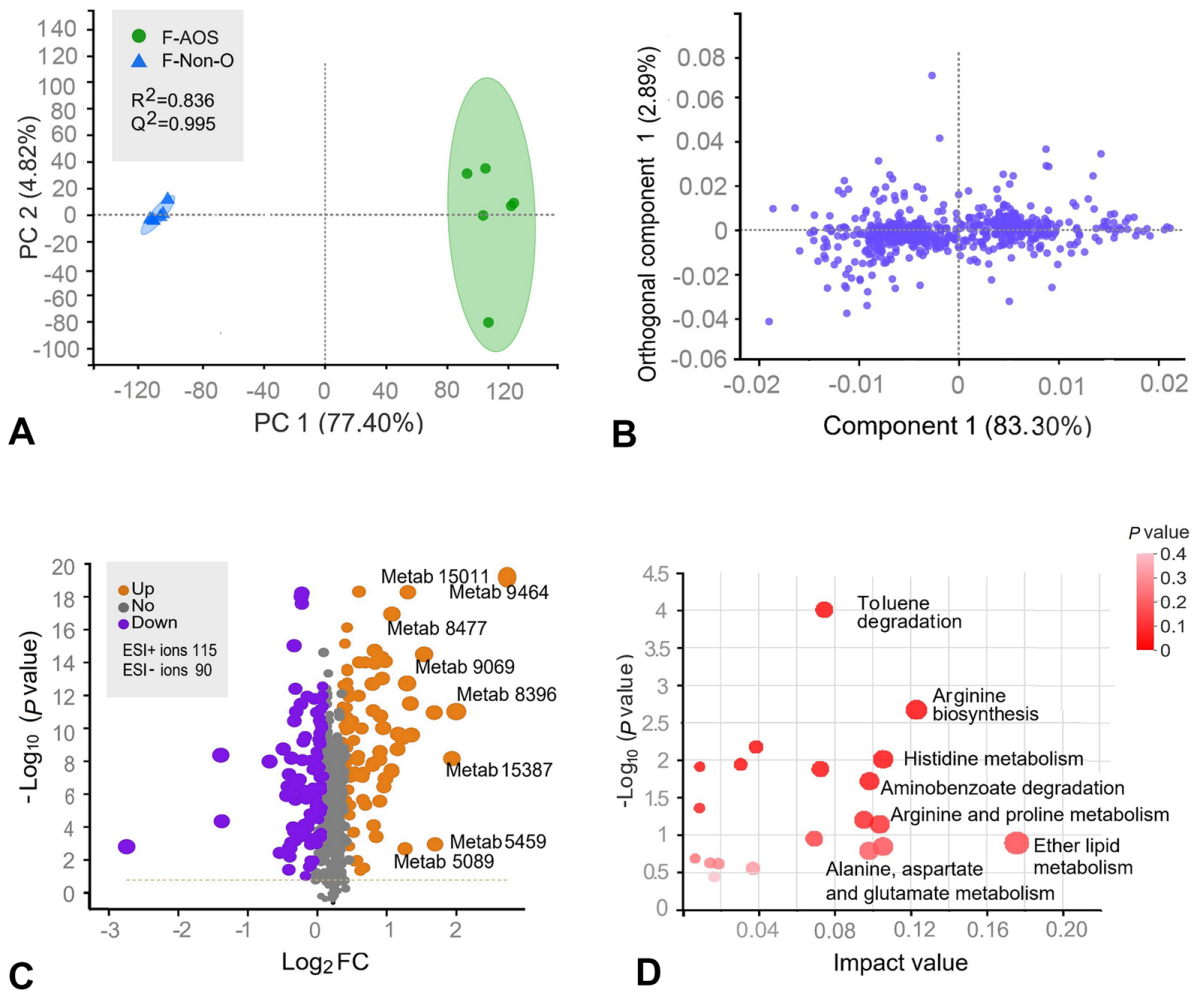
Effects of AOS supplementation (F-AOS) on fecal microbiota metabolomics. The medium without adding any oligosaccharides was used as the control (F-Non-O). **A** PCA score plot. **B** OPLS-DA score plot. **C** The volcano plot was used to visualize differences between groups with yellow dots representing distinct upregulated metabolites, purple dots representing downregulated metabolites, and grey dots representing non-significant differences. **D** Bubble diagram of enriched KEGG pathways. Sizes of dots represent number of metabolites

**Fig. 6:**
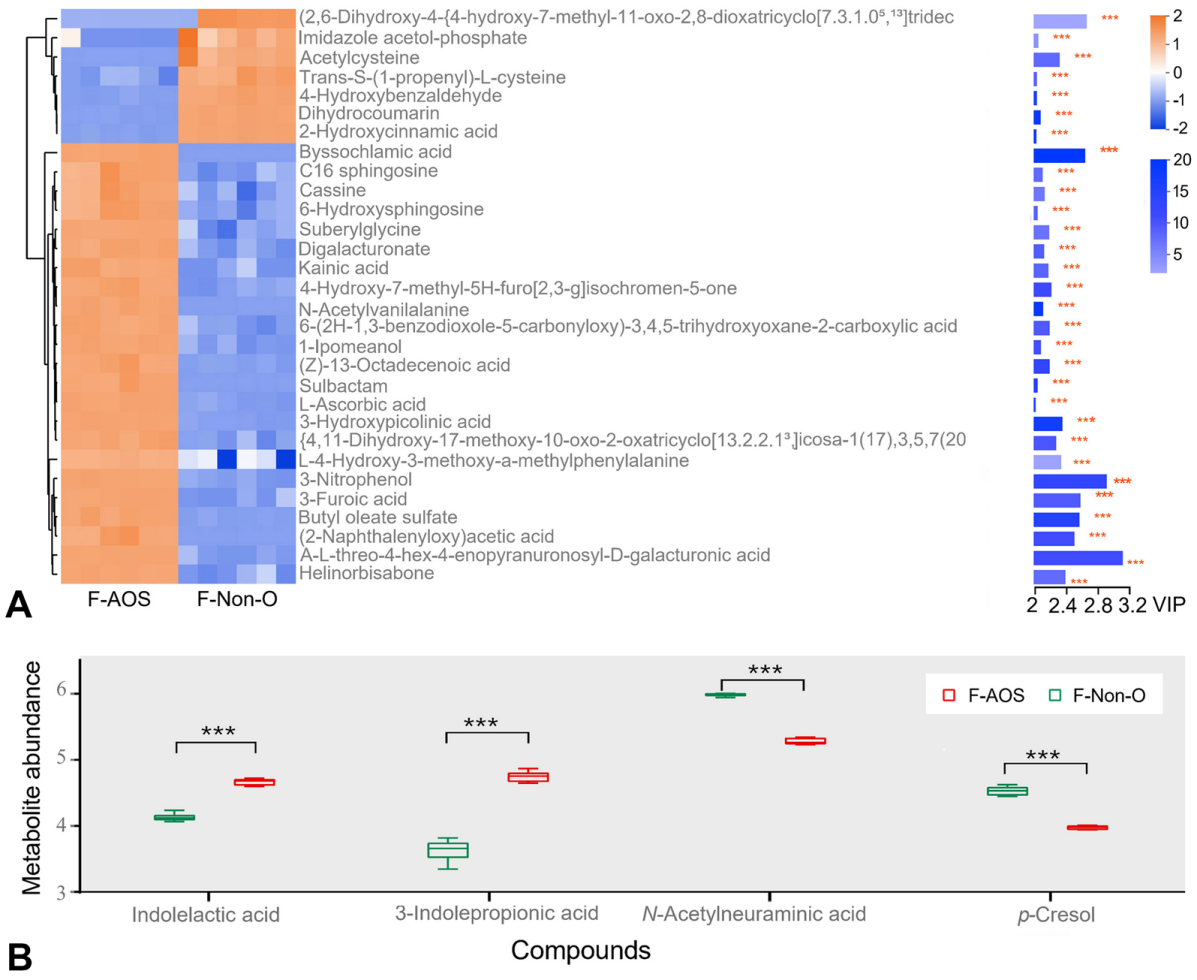
Metabolite analysis of AOS supplementation (F-AOS) on fecal microbiota metabolomics. The medium without the addition of oligosaccharides was used as the control (F-Non-O). **A** VIP scores analysis. Heatmap with yellow or blue boxes on the left indicates high and low abundance ratio, respectively. **B** The abundance ratio of selected potential metabolites biomarkers. Statistical significance was determined by Student’s *t*-test (*n* = 6). ***, *P* < 0.001

F-AOS treatment inhibited growth and downregulated key genes involved in flagellar assembly and the type III secretory system of *S*. Typhimurium. Therefore, differences in metabolite composition between the F-AOS and F-Non-O groups were studied using untargeted metabolomics. PCA showed that the F-AOS and F-Non-O groups were clearly separated in the PC1 × PC2 score plots, with PC1 exhibiting a variance of 77.40% and Q^2^ value of 0.995 (Fig. 5A). Orthogonal partial least squares discriminant analysis (OPLS-DA) score plots exhibited similar statistical distribution patterns with a total variance of 86.19% (Fig. 5B). The volcano plot of the metabolite content indicated significant differences between the two groups (Fig. 5C, Supplementary Table S4). Of the 736 identified metabolites, 205 were markedly different in the F-AOS group compared to those in the F-Non-O group; the numbers of ESI + and ESI- ions were 115 and 90, respectively (Supplementary Table S4). Metabolic pathway enrichment analysis based on the KEGG database was conducted using MetaboAnalyst (Fig. 5D). Metabolites with significantly increased concentrations were enriched in ether lipid metabolism (map00565), toluene degradation (map00623), and amino acid metabolism pathways, including arginine biosynthesis (map00220), arginine and proline metabolism (map00330), histidine metabolism (map00340), alanine, aspartate, glutamate metabolism (map00250), and aminobenzoate degradation (map00627). Purine metabolism was the most significantly enriched pathway (-log_10_ *p* = 4.01), in which three metabolites (adenine, xanthine, and l-glutamine) were downregulated and two metabolites (5-aminoimidazole ribonucleotide and adenosine phosphosulfate) were upregulated in the F-AOS group. To present the differences in metabolite content, metabolites exhibiting significant changes in levels were selected to construct a heat map (Fig. 6A). Thirty-one (31) metabolites had variable importance in projection (VIP) scores > 2.0, including 2-naphthalenyloxy acetic acid (VIP = 2.50), 3-hydroxypicolinic acid (VIP = 2.35), suberylglycine (VIP = 2.18), and C16 sphingosine (VIP = 2.01). According to the OPLS-DA analysis, the levels of potential biomarkers significantly changed in the F-AOS group compared to those in the F-Non-O group (*P* < 0.001, Fig. 6B). AOS enhanced the concentration of indolelactic acid and 3-indolepropionic acid, suggesting AOS fermentation promoted tryptophan metabolism. In addition, decreased levels of *N*-acetylneuraminic acid (Neu5Ac) and *p-*cresol were observed in the F-AOS group compared to those in the control group (*P* < 0.001).

### Detection of pathogenicity-related genes and potential metabolites as biomarkers

**Fig. 7:**
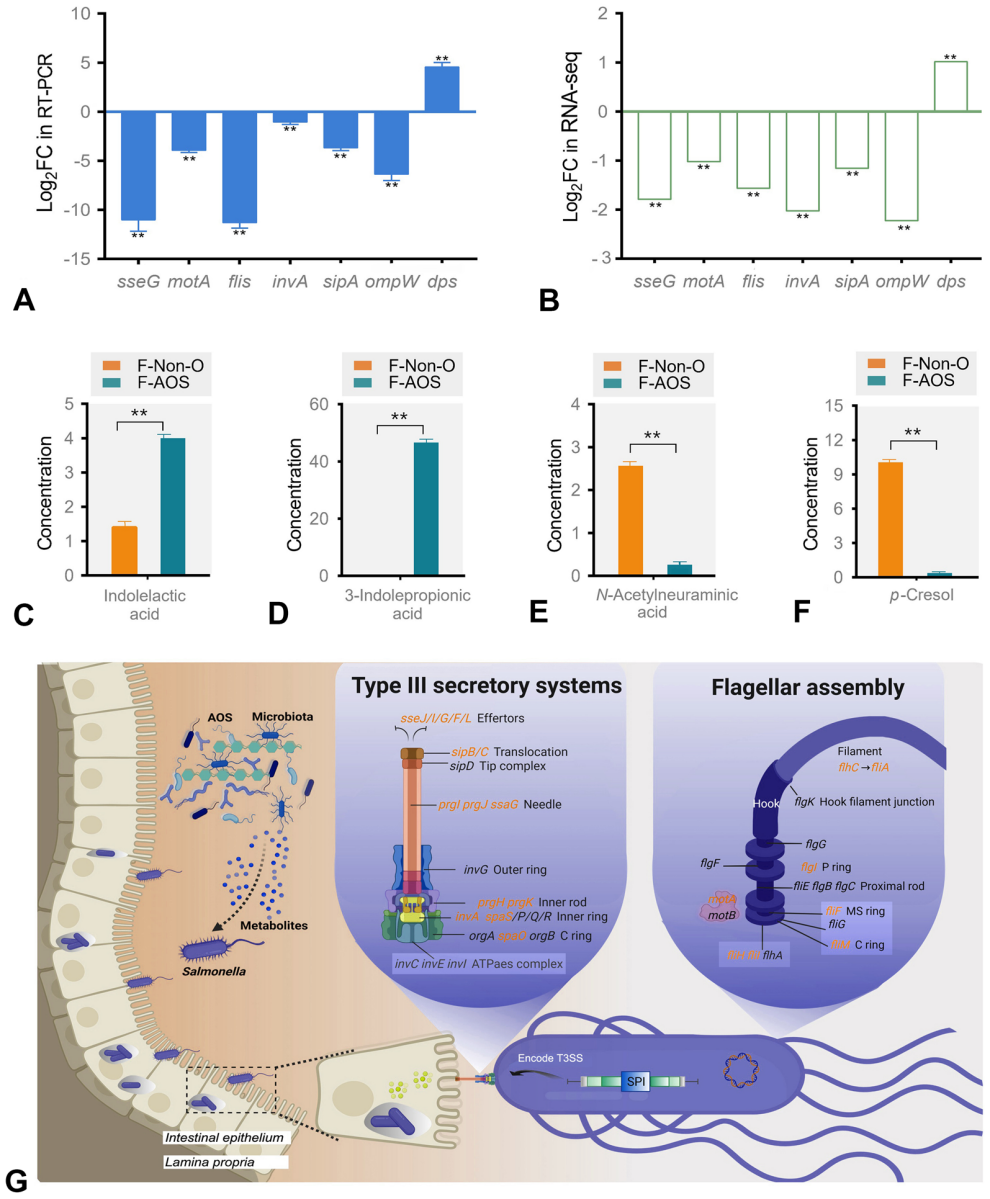
Integrated transcriptomic and metabolomic analysis of inhibition effect of F-AOS against *S*. Typhimurium. **A** RT-qPCR results of seven DEGs. The expression of each gene was normalized to the average expression of the endogenous reference gene 16S rRNA. **B** RNA-seq results of the DEGs. Quantification of potential biomarkers of indolelactic acid **C**, 3-indolepropionic acid **D**, *N*-acetylneuraminic acid **E**, and* p*-cresol **F** in F-AOS by HPLC. **G** Possible responses of *S*. Typhimurium to F-AOS. Red colors represent downregulated at the mRNA level. F-AOS, supernatant of AOS fermented with chicken fecal inoculum. Supernatant of non-O fermented (F-Non-O) was used as the control. Statistical significance was determined by Student’s *t*-test (*n* = 3). **, *P* < 0.01

An integrated analysis of differential metabolites (VIP > 1, *P* < 0.05) and DEGs (log_2_ FC > 1 or < -1, *q* value < 0.05) was performed to explore the altered mRNA levels in *S*. Typhimurium in response to F-AOS treatment (Fig. 7). To validate the results of RNA-seq analysis, RT-qPCR was performed to examine the mRNA expression of significant functional DEGs involved in the type III secretion system (*sseG*, *invA*, and *sipA*), flagellar assembly (*motA* and *fliF*), and several key virulence genes (*ompW* and *dps*). The mRNA expression analysis validated that F-AOS downregulated the expression levels of *sseG*, *motA*, *fliF*, *invA*, *sipA*, and *ompW* and upregulated that of *dps* compared to those in the F-Non-O group (*P* < 0.01). The expression trends of the seven DEGs were similar to those obtained by RNA-seq, suggesting RNA-seq data reliably reflected the gene expression trends (Fig. 7A, B).

F-AOS exhibited higher inhibitory effects against *S*. Typhimurium than AOS during single-strain cultivation, showing novel metabolites with antibacterial activity were produced during fermentation of AOS by fecal microbiota. In this study, four metabolites were quantified to elucidate the antibacterial mechanism of AOS in the fermentation liquid of intestinal microbiota. F-AOS treatment exhibited increased production of indolelactic acid and 3-indolepropionic acid, with the contents of 4.00 and 45.60 μg/mL, respectively (Fig. 7C, D). By contrast, the F-Non-O group showed low production of indolelactic acid (1.20 μg/mL) and almost undetectable levels of 3-indolepropionic acid. However, the F-Non-O group exhibited a higher content of Neu5Ac (2.56 μg/mL) than the F-AOS group (0.26 μg/mL) after fermentation (Fig. 7E, F). A significant difference was observed in the production of *p*-cresol (*P* < 0.01).

Based on integrated transcriptomic and metabolomic analyses, the metabolites in F-AOS were hypothesized to affect the gene expression of *S*. Typhimurium, especially the genes involved in flagellar assembly and type III secretory system pathways (Fig. 7G). AOS enhanced the abundance of key functional metabolites, including indolelactic acid and 3-indolepropionic acid, during in vitro chicken fecal fermentation, which might promote intestinal development and modulate microbiota composition.

## Discussion

AOS as the oligosaccharides extracted from brown algae, are a promising feed additive for the breeding industry. Enzymatically prepared AOS exhibited prominent inhibitory activity against *Vibrio* infections in shrimp by enhancing the immune functions of the host (Jiang et al. 2017). Moreover, enzymatically prepared AOS alleviated mortality in broilers caused by *Salmonella enteritidis* infection and enhanced mucosal cytokine expression and antibody production (Yan et al. 2011). AOS disrupt biofilm formation by binding to bacterial surfaces, modulating surface charges, inducing microbial aggregation, and inhibiting motility (Powell et al. 2014). In the present study, the inhibitory effects of AOS and F-AOS against *S*. Typhimurium in the intestinal microbiota of chickens were investigated. Notably, AOS did not directly inhibit *S*. Typhimurium growth in single-strain cultivation, which is different from the previous study. Hu et al. (2005) found that enzymatically prepared AOS exerted inhibitory effects on *Salmonella *in vitro, with minimum inhibitory concentrations of 0.225 μg/mL. The previous study also did not explain the mechanism by which AOS directly inhibited *S*. Typhimurium. Interestingly, F-AOS exhibited remarkable antibacterial activity.

The present study revealed that AOS were slowly utilized and fermented by chicken intestinal microbiota, indicating that AOS, as acidic oligosaccharides, ferment slower than prebiotics which can be rapidly fermented. Most prebiotics are neutral oligosaccharides, such as GOS and fructo-oligosaccharides. The slow fermentation of AOS was crucial for the reconstruction of distal intestinal microbiota and the improvement of intestinal health (Li et al. 2016). In this test, the molecular weight of AOS was 1.93 kDa, a little higher than that of GOS (< 1 kDa), which might influence its fermentation rate. However, the presence of uronic acid is the most important reason for the slow utilization rate of AOS.

F-AOS exhibited a notable inhibitory effect against *S*. Typhimurium, reducing the abundance ratio of *S*. Typhimurium in chicken intestinal microbiota. In the experiments on the antibacterial activity of AOS against *S*. Typhimurium in chicken fecal cultures, the abundance ratio of *S*. Typhimurium in the control group was significantly higher than that in the AOS-treated group, indicating AOS inhibited the proliferation of *S*. Typhimurium in the chicken gut microbiota. Moreover, the antibacterial activity of F-AOS against *S*. Typhimurium during single-strain cultivation was elucidated. F-AOS exhibited a significantly lower OD_600_ (0.45) than F-Non-O at 48 h (0.59; *P* < 0.05). Similarly, in a previous study, the in vitro fermentation of AOS using pig feces resulted in a decreased abundance of opportunistic pathogenic bacteria, such as *Escherichia*, *Shigella*, and *Peptoniphilus* (Han et al. 2019). To further verify how AOS inhibit *S*. Typhimurium through the metabolism of the gut microbiota, we carried out subsequent experiments.

RT-qPCR analysis revealed that AOS reduced *ompW* expression levels in *S*. Typhimurium. *OmpW* encodes a minor porin involved in osmoregulation and participates in the adaptation of the bacterium to diverse environments, drug resistance mechanisms, and bacterial pathogenesis (Gil et al. 2009). AOS with specific M/G ratio exhibited penetration effect on bacterial cells (Powell et al. 2013), which may be related to the mechanism by which AOS reduced *ompW* expression levels. F-AOS exerted a remarkable inhibitory effect on *S*. Typhimurium growth, which could be attributed to the metabolites produced during AOS fermentation in the intestinal microenvironment. As prebiotics, oligosaccharides have important industrial value for improving animal health and reducing antibiotic abuse in the breeding industry. At present, the functions of oligosaccharides are usually focused on increasing the abundance of beneficial intestinal bacteria and the content of their metabolites (Li et al. 2020a, b). These metabolites are absorbed across the host gut and promote health (Krautkramer et al. 2021). Enzyme-treated soybean meal increased the fecal butyrate level, average daily gain, and relative abundance of *Bacteroidetes*, *Oscillospiraceae*, and *Christensenellaceae* (Zhang and Piao 2022). Dietary fibers in mung bean coats increased the abundance of beneficial bacteria, promoting the synthesis of SCFAs (Xie et al. 2022). In this study, because of the metabolism of AOS, fecal microbiota inhibited the growth of *S*. Typhimurium and the expression of pathogenicity-related genes, which provides a new idea for the development of marine oligosaccharides as feed prebiotics.

F-AOS exerted a significant inhibitory effect on the transcription of type III secretory system genes and expression of effector proteins in *Salmonella* flagella. *S*. Typhimurium causes severe diseases in chickens, leading to public health issues and significant economic losses (LaRock et al. 2015). *S*. Typhimurium uses multiple virulence factors to overcome colonization resistance and induce intestinal inflammation (Kaiser et al. 2012). After entering the intestinal lumen, *S*. Typhimurium moves close to the intestinal epithelial cells and attaches to the cell surface using flagella and fimbriae; the fimbriae bind to the glycoprotein laminin of the extracellular matrix and mediate adhesion to the host cells. The autotransporter protein MisL binds to fibronectin and *S*. Typhimurium adhesins (SiiE and BapA), allowing the bacteria to tightly adhere to the intestinal epithelium (Fàbrega and Vila 2013). In this study, the expression of adhesins and autotransporter proteins was slightly affected by F-AOS treatment; the expression levels of flagellar proteins encoded by *motA*, *flg*, *flhA*, *flhC*, *flhD*, *fliF*, *fliH*, *fliI*, *fliM*, and *fliS* were significantly lower in the F-AOS group than in the control group (*P* < 0.05). Chevance and Hughes (2008) described the mechanism of flagellar assembly in *Salmonella* mainly through a three-class hierarchy. In this study, *flhDC*, of class I hierarchy operon gene, was significantly downregulated by F-AOS (*P* < 0.05). Moreover, the expression levels of *fliF*, *flgI*, *flhA*, and *fliS* genes involved in the class II hierarchy, required for the structural maintenance and assembly of the flagellar motor, were also downregulated (*P* < 0.05). The expression levels of *fliH* and *fliI*, which encode components of the type III injectisome apparatus, were also decreased (*P* < 0.05). Moreover, the expression levels of genes encoding flagellar biosynthesis protein (*flhA*) and flagellar export chaperone (*fliS*) were reduced by 1.09 log_2_ FC and 1.20 log_2_ FC, respectively. The expression of flagellar rotation protein (MotB) was also significantly downregulated in the F-AOS group, which reduced the ability of the bacteria to produce biofilms. Thus, F-AOS possibly disrupted flagellar assembly and blocked the attachment of *S*. Typhimurium to the gut cell surface.

Virulence genes of *S*. Typhimurium are clustered on pathogenic islands (SPIs) (Kaur and Jain 2012). *S*. Typhimurium pathogenicity islands 1 (SPI-1) and 2 (SPI-2) encode two type III secretory systems (T3SS), which are syringe-like apparatuses used to translocate bacterial proteins into host cells. In this study, most of the proteins in the T3SS, including those encoded by *sseF*, s*saG*, p*rgI*, *prgJ*, *sseJ*, *sseI*, s*opD2*, *pipB2*, *sspH2*, *sseL*, *sipC*, *sopE2*, s*ipA*, *sseG*, and *gapA*, which are essential for bacterial survival in host cells and are key factors in determining bacterial virulence, were downregulated. The decrease in *ssaG* and *sipA* expression levels was further verified using RT-qPCR (*P* < 0.05). SPI-1 T3SS (T3SS-1) is associated with epithelial cell invasion. *S*. Typhimurium injects effector proteins encoded by *sipA*, *sopA*, *sopB*, *sopD*, and *sopE2* via a needle into the host cell, where it triggers cytoskeletal rearrangement and bacterial engulfment (Notti and Stebbins 2016). The T3SS-1-secreted protein effector SopE2 is required for invasion, whereas SipA accelerates entry into cells. SipA and SopE2 contribute to the invasion of epithelial cells by *S*. Typhimurium in vitro (Raffatellu et al. 2005). T3SS-2 contributes to the spread of bacterial and systemic infections. Here, F-AOS treatment downregulated the expression levels of *sopD2*, *pipB2*, *sseJ*, and s*seF* genes encoding six effectors secreted by T3SS-2, which manipulate host cell intracellular trafficking and establish an intracellular replicative niche for *S*. Typhimurium. In the *sse* family, *sseA* and *sseJ* are closely associated with bacterial survival and virulence (Drecktrah et al. 2004; Zurawski and Stein 2003). The gene *sseI* is highly similar to *sspH2* of SPI-2 effectors, inhibiting normal host cell migration and ultimately counteracting the ability of the host to clear systemic bacteria. F-AOS treatment downregulated the expression of genes of the *sse* family by reducing the transcription of *sseF*, *sseJ*, *sseI*, *sseL*, and *sseG* (*P* < 0.05).

To further elucidate the molecular mechanism of F-AOS, untargeted metabolomics was used to identify potential functional metabolite biomarkers in F-AOS. AOS significantly enhanced the abundance of tryptophan metabolism biomarkers, indolelactic acid and 3-indolepropionic acid. The gut microbiota influences host metabolism and immunity through metabolic activities, such as the production of tryptophan metabolites, which activate the immune system, enhance the intestinal epithelial barrier, and selectively modulate the gut microbiota composition (Wikoff et al. 2009). Moreover, species such as *Clostridium sporogenes*, *Peptostreptococcus russellii*, *Pasteurella stomatis*, and *Peptostreptococcus anaerobius* produce indolepropionate and indoleacrylate. Many studies have shown that 3-indolepropionic acid inhibits endotoxin leakage in rats, correlates with intestinal barrier homeostasis, and regulates multiple inflammatory cytokines (Venkatesh et al. 2014). Substantial amounts of indolelactic acid in the gut are associated with the capacity to activate the aryl hydrocarbon receptor, which enhances the gut barrier function, protects against pathogenic infections, and influences host metabolism (Laursen et al. 2021).

AOS significantly decreased the levels of potentially harmful biomarkers of host metabolism, including *p-*cresol and Neu5Ac. It is believed that *p-*cresol is a phenolic metabolite that decreases the integrity of the gut epithelium and reduces the proliferation and viability of intestinal epithelial cells (Andriamihaja et al. 2015). It is produced by many gut bacterial species, including members of the family *Enterobacteriaceae* and *Clostridium* clusters I, XI, and XIV (Saito et al. 2018). Invasion by pathogenic bacteria alters nutrient types and concentrations and increases competition with the endogenous intestinal microbiota (Jeong et al. 2009). Neu5Ac acts as a nutrient for enteropathogenic bacteria when they invade and colonize the gut (Steenbergen et al. 2009).

In summary, AOS did not inhibit the growth of *S*. Typhimurium during in vitro single-strain cultivation. The remarkable antibacterial effect of F-AOS against *S*. Typhimurium was confirmed, indicating that AOS may affect fecal microbiota metabolism to inhibit pathogen growth. Moreover, F-AOS downregulated the mRNA expression of key genes involved in flagellar assembly and the T3SS in *S*. Typhimurium, illustrating that AOS could interfere with its infecting ability indirectly by modulating the gut microbiota metabolism. In addition, AOS increased the metabolite concentrations of indolelactic acid and 3-indolepropionic acid and decreased those of Neu5Ac and *p*-cresol, thereby contributing to intestinal development and resistance against pathogens. Thus, this study provides novel insights into the antibacterial mechanism of AOS against common enteric pathogenic bacteria and indicates that AOS, with an Mw of 1.93 kDa, may be considered an efficient antibiotic substitute in poultry feed. However, a more detailed understanding of the inhibitory mechanisms of tryptophan metabolites against *S*. Typhimurium is required.

## Materials and methods

### Materials

*Salmonella enterica* subsp. *enterica* serovar Typhimurium strain CMCC 50,222 was purchased from the National Center for Medical Culture Collections (http://www.cmccb.org.cn/). The chromatographical-grade standards including indole-3-carboxaldehyde, 3-indolepropionic acid, indolelactic acid, *p-*cresol, and Neu5Ac were all purchased from Shanghai Macklin Biochemical Co., Ltd. (Shanghai, China). Mannuronate oligosaccharides (DP 1–6) and guluronate oligosaccharides (DP 2–6) were purchased from Qingdao Bz Oligo Biotech Co., Ltd. (Qingdao, China). Alginate was purchased from Sinopharm Chemical Reagent Beijing Co., Ltd. (Beijing, China). GOS (Mw < 1 kDa, purity ≥ 90%) was purchased from New Francisco Biotechnology Co., Ltd. (Yunfu, China). Other chemicals and reagents were of analytical grade. Luria–Bertani (LB) medium, deoxycholate hydrogen sulfide lactose (DHL) agar, and brain heart infusion (BHI) agar were purchased from Qingdao Hope Bio-Technology Co., Ltd. (Qingdao, China).

AOS were enzymatically prepared from alginate according to previously reported method (Yang et al. 2018). In brief, 5% (w/v) alginate was mixed with the alginate lyase AlyM (70 U/mL, provided by Applied Microbiology Lab., Ocean University of China) and incubated at 45 ℃ for 6 h. The final enzymatic hydrolysates were sterilized for enzyme inactivation at 90 ℃ for 15 min and centrifuged at 10,000 *g* for 10 min in a refrigerated centrifuge (Thermo Fisher Scientific, Waltham, MA, USA) to collect the supernatant. High-Mw fractions in supernatant were precipitated to remove using fivefold ethanol and AOS were finally collected after a centrifugation at 5000 *g* for 10 min followed by freeze-dried. The prepared AOS have Mw of 1.93 kDa and M/G ratio of 0.37.

### AOS fermentation by chicken fecal microbiota in vitro

#### Collection of chicken feces

Freshly dropped excreta of paste-like texture without urine were predominantly collected. Within 5 to 10 min after defecation, feces were aseptically collected and kept under anoxic conditions in sterile and airtight tubes, and then immediately moved to a YQX-T anaerobic incubator (Longyue Instrument Equipment Co., Ltd., Shanghai, China) in the laboratory. Fecal slurries were prepared by diluting the samples at a ratio of 1:5 (w/v) in sterile anaerobic phosphate buffer (0.01 mol/L, pH 7.0) containing 1 g/L cysteine hydrochloride as reducing agent. Slurries were filtered to remove large particles using a multi-layer gauze then sealed in anaerobic serum bottles for further inoculation.

#### In vitro fecal fermentation

The in vitro fecal fermentation media were prepared as previously described (Xiao et al. 2022). The fecal filtrate was supplemented into the anaerobic bottles containing 50 mL medium with an inoculation amount of 5% (v/v). AOS, GOS, and glucose were added as the sole carbon sources, respectively, with a final concentration of 15 mg/mL, and the medium without adding any oligosaccharides was set as the control. The fermentation samples in all groups were collected at 0, 24, 48, 72, 96, 120, and 144 h, respectively, and centrifuged at 13,000 *g* and 4℃ for 10 min. The supernatant was collected for the determination of pH variation and carbohydrate utilization.

#### Determination of pH variation and carbohydrate utilization

The pH values and OD_600_ were determined using LAQUAtwin-pH-22 (Horiba Stec Co., Ltd., Tokyo, Japan) and 96-well microtiter plates (Thermo Fisher Scientific, Waltham, MA, USA). Carbohydrate utilization was characterized by TLC (silica gel 60 F254 plate, Merck, Darmstadt, Germany). The mobile phase used for AOS and non-O groups was N-butyl alcohol/formic acid/distilled water (4:6:1, v/v/v), and for GOS and glucose groups was N-butanol/acetic acid/water (1:1:1, v/v/v). The chromogenic agent was prepared by dissolving 1 mL HCl, 2 mL aniline, 2 g diphenylamine, and 10 mL 85% H_3_PO_4_ in 100 mL acetone.

#### Detection of AOS antibacterial activity against *S*. Typhimurium in chicken fecal cultures

To preliminarily determine whether AOS can inhibit *S*. Typhimurium in intestinal microbiota, *S*. Typhimurium was added into the chicken feces with the addition amount of 10^4^–10^5^ CFU/g. The in vitro fecal fermentation was performed as the previous method. Fecal samples in the media without adding AOS were set as the control. For identification and enumeration of *S*. Typhimurium, the precipitated bacteria were diluted to density gradient and spread on DHL agar plates and BHI agar plates. The medium was incubated under anoxic condition at 37 °C for 24 h. The *S*. Typhimurium and total bacteria colonies were counted, respectively. The relative abundance of *S*. Typhimurium was performed using following formula, and all analyses were performed in triplicate.

Abundance ratio (%) = *S*. Typhimurium abundance (CFU/mL)/total bacteria abundance (CFU/mL).

### Antibacterial mechanism of AOS against *S*. Typhimurium

#### Direct antibacterial activity of AOS during single *S*. Typhimurium cultivation

*S*. Typhimurium was inoculated in the LB medium containing 15 g/L AOS, with the inoculation amount of 5% (v/v). The LB medium without AOS was used as the control. Bacterial growth was assessed by monitoring the pH and OD_600_. The *sipA*, *invA*, and *ompW* genes, related to adhesion and colonization of *S*. Typhimurium, were selected for indicating virulence genes expression using RT-qPCR, with 16S rRNA as a housekeeping reference gene. Primers used to quantify these genes are listed in Supplementary Table S1. Total RNA was extracted from the bacterial precipitation using TRIzol® Reagent Invitrogen (Life Technologies, Carlsbad, CA, USA) according to the manufacturer instructions and genomic DNA was removed using DNase I (Vazyme, Nanjing, China). The cDNA synthesis and gene amplification were performed following previously reported methods (Liang et al. 2022).

#### Antibacterial effect of supernatant of fermented-AOS during single *S*. Typhimurium cultivation

The supernatant of AOS fermented and non-O fermented with chicken fecal inoculum cultures were prepared. The *S*. Typhimurium was activated in LB medium before inoculation. *S*. Typhimurium solution (1 mL, OD_600_ = 0.5) was added to the 16 mL LB medium, and subsequently 4 mL of F-AOS and F-Non-O were supplemented, respectively. The culture in all groups was collected at 0, 24, 48, 72, 96, 120, and 144 h, respectively, and OD_600_ was determined.

Transcriptomic analysis was adopted to explain the response mechanism of *S*. Typhimurium to F-AOS (48 h of fermentation). Total RNA extraction of *S*. Typhimurium was the same as previous described. RNA-seq library was prepared following the TruSeqTM RNA sample preparation Kit from Illumina (San Diego, CA, USA) using 2 μg of total RNA. After being quantified by TBS380, the paired-end RNA-seq sequencing library was sequenced with the Illumina HiSeq × TEN (2 × 150 bp read length). Gene expression level was calculated using fragments per kilobase of exon model per million mapped reads FPKM. DEGs were screened by DESeq2 with a filter criterion of FC > 2 or < -2 and *q* value < 0.05. R statistical package software EdgeR (empirical analysis of digital gene expression in R) was used for the differential expression analysis. GO functional enrichment and KEGG pathway enrichment analyses were carried out by Goatools and KOBA 2.1.1.

Virulence genes that contribute to the toxicity and adhesion of *Salmonella* were selected based on the RNA-seq data for further validation using RT-qPCR. The 16S rRNA was used as a housekeeping reference gene. Primers are listed in Supplementary Table S1. RNA extraction and gene amplification were the same as previous described.

### Untargeted metabolomic profile of F-AOS

#### Metabolites extraction and analysis

F-AOS and F-Non-O samples at 96 h of fermentation, collected as previous mentioned, were thawed on ice. A quality control (QC) sample was made by mixing and blending equal volumes (10 μL) of each fermentation sample and injected at regular intervals (every eight samples). Metabolites in F-AOS and F-Non-O were extracted with methanol/water (4:1, v/v) solution. The samples were placed at − 20℃ for 30 min to precipitate proteins. After centrifugation at 13000 *g* and 4℃ for 15 min, the supernatant was carefully transferred to sample vials for LC–MS/MS analysis. Samples (10 μL) were injected into ExionLCtmad system (AB Sciex, Milford, MA, USA) equipped with an Acquity UPLC Beh C18 column (100 mm × 2.1 mm, 1.7 µm; Waters, Milford, MA, USA). The instrumental and chromatographic conditions were as reported by Liu et al. (2020).

#### Metabolic data processing method

Metabolic data processing was conducted according to the method of Bai et al. (2021) with modifications. The metabolites with VIP value > 1 and *P* < 0.05 are regarded as the potential biomarkers for the differentiation between F-AOS and F-Non-O. PCA and OPLS-DA were used to profile differentiation between two groups. Experiments were performed with six replicates and significant differences were shown at *P* < 0.05. The model validity was evaluated from model parameters R^2^ and Q^2^, which provide information for the interpretability and predictability, respectively, of the model and avoid the risk of over-fitting. Differential metabolites among two groups were summarized and mapped into their biochemical pathways through metabolic enrichment and pathway analysis based on a database search (KEGG, http://www.genome.jp/kegg/).

### Major metabolites analysis by HPLC

Indolelactic acid and 3-indolepropionic acid contents were determined using Agilent 1260 Series HPLC system equipped with Zorbax SB-Aq column (3.0 mm × 150 mm, 1.8 μm; Agilent Technologies, Santa Clara, CA, USA) at 35℃ with a UV detection wavelength of 275 nm. The mobile phase was 0.1% trifluoroacetic acid/methanol (90:10, v/v) at a flow rate of 2 mL/min, and the injection volume was 20 µL. Chromatography grade indolelactic acid and 3-indolepropionic acid were used as standards, and indole-3-carboxaldehyde was used as the internal standard.

Neu5Ac was measured using UV detection with a wavelength of 230 nm as described previously with slight modifications (Zhao et al. 2021). Zorbax 300 XDB-C18 column (4.6 mm × 250 mm, 5 μm; Agilent Technologies, Santa Clara, CA, USA) was used in HPLC. Column temperature was maintained at 35 ℃. The mobile phase was acetonitrile/water (10:90, v/v) at a flow rate of 1.0 mL/min. The standard calibration curve was developed by plotting the peak areas of Neu5Ac against the set of concentrations used for standard preparation (*y* = 33411 *x*–241.49, *R*^2^ = 0.9991).

The *p-*cresol analysis was conducted according to the method of Singh with slight modifications (Singh et al. 2017). Zorbax 300 XDB-C18 column accompanied with UV detector (measuring absorbance of 275 nm) was used in HPLC. The mobile phase was methanol/water (60:40, v/v) at a flow rate of 0.7 mL/min. The quantification of the compounds was based on the absorbances of known quantities of *p-*cresol (*y* = 12765*x* + 86.623, *R*^2^ = 0.9993).

### Statistical analysis

Statistical analysis was performed using SPSS 21.0 (SPSS, Chicago, IL, USA) and figures were prepared using GraphPad Prism 8.02 software (GraphPad, La Jolla, CA, USA) and BioRender (https://biorender.com/, Toronto, Canada). Statistical significance between two groups was processed by Student’s *t*-test. Difference was considered significant when *P* < 0.05.

## Supplementary Information

Below is the link to the electronic supplementary material.

Supplementary file1 (DOCX 540 KB)

Supplementary file2 (XLSX 159 KB)

## Data Availability

The datasets generated and analyzed during the current study are available from the corresponding author on reasonable request.
