# Supplementary material for: Response of Salmonella enterica serovar Typhimurium to alginate oligosaccharides fermented with fecal inoculum: integrated transcriptomic and metabolomic analyses
Source: Mar Life Sci Technol. 2023 May 29;5(2):242–56. doi: 10.1007/s42995-023-00176-z (PMC10232696; doi:10.1007/s42995-023-00176-z)
Supplement: Supplementary file 1 — Supplementary file1 (DOCX 540 KB) [file 42995_2023_176_MOESM1_ESM.docx]

Supporting Information

Marine Life Science & Technology

Response of *Salmonella enterica* serovar Typhimurium to alginate oligosaccharides fermented with fecal inoculum: integrated transcriptomic and metabolomic analyses

Jiaying Cheng, Mengshi Xiao, Xinmiao Ren, Francesco Secundo, Ying Yu, Shihao Nan, Weimiao Chen, Changliang Zhu, Qing Kong, Youtao Huang, Xiaodan Fu*****, Haijin Mou*****

***Corresponding Author**

**Xiaodan Fu**

Assistant research fellow

State Key Laboratory of Food Science and Technology, Nanchang University

No. 235 Nanjing East Road, Nanchang 330047, Jiangxi Province, China

EMAIL: luna_9303@163.com

**Haijin Mou**

College of Food Science and Engineering, Ocean University of China

No. 5 Yushan Road, Qingdao 266003, Shandong Province, China

EMAIL: [mousun@ouc.edu.cn](mailto:mousun@ouc.edu.cn)

TEL: 86-532-82032290

**
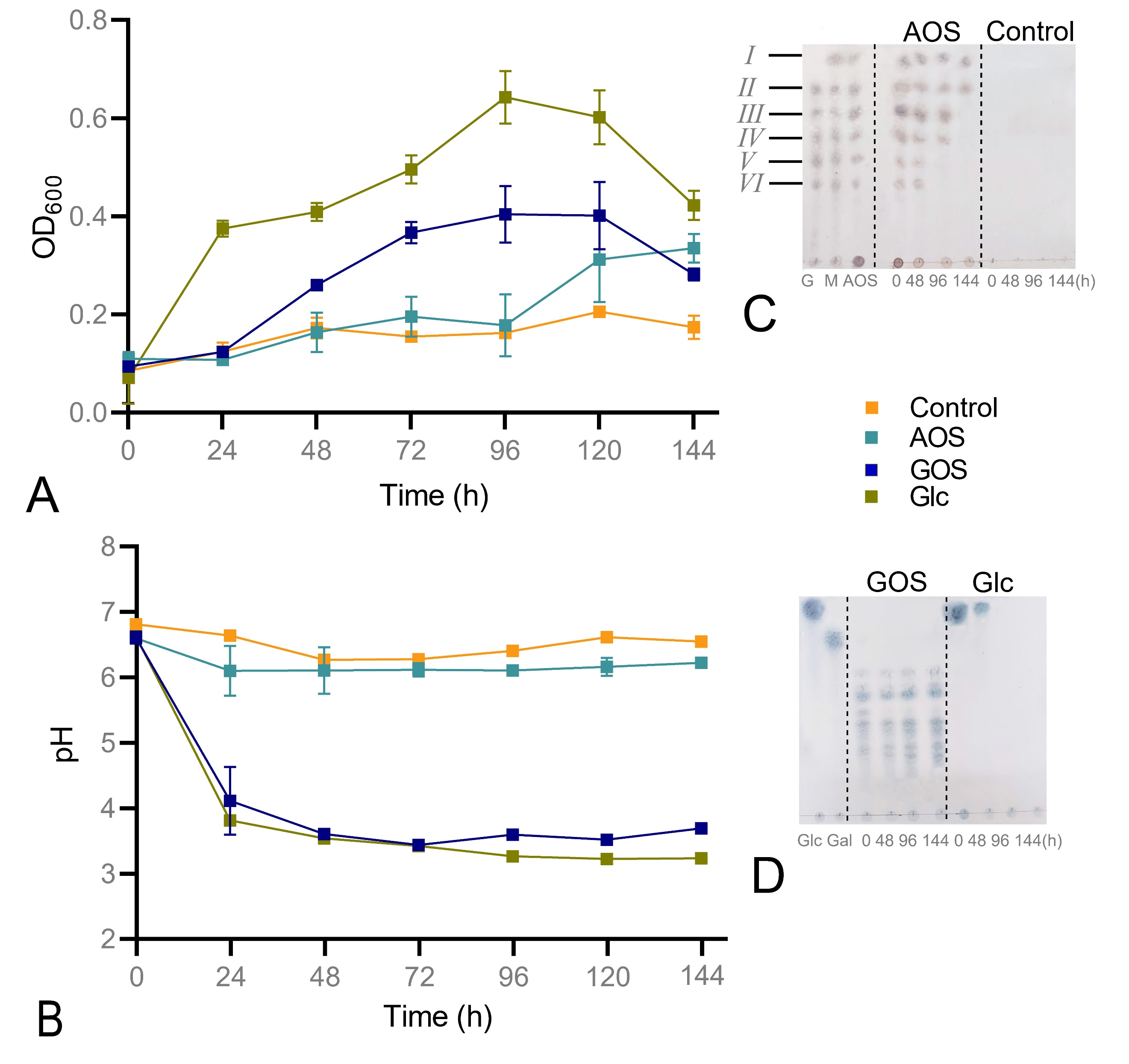
**

**Fig. S1** *In vitro* fermentation profile of AOS, GOS, and glucose by chicken fecal microbiota. (A) Growth curves of chicken fecal microbiota. (B) Changes of pH values. TLC profiles of degradation of AOS (C), GOS, and glucose (D) during *in vitro* fermentation. Control, without adding any oligosaccharides. Glc, glucose; Gal, galactose; GOS, galacto-oligosaccharide; M, mannuronate oligosaccharides (DP 1-6); G, guluronate oligosaccharides (DP 2-6); *I*-*VI*, DP 1-6. n = 3

**Supplementary Table 1**

**Table S1 Primers sequences for RT-qPCR**

| Primer ID | Primer Sequence |
| --- | --- |
| *sseG*5' | ACCTGTTAGCCCAAATGC |
| *sseG*3' | CTCTATCACTCGTTTCGCAA |
| *motA*5' | CTGCTGGTTTGGGTTTCT |
| *motA*3' | GTCTAATCTGAACGGCTATGC |
| *fli*s5' | GGTGAGATTGCGACGAAT |
| *flis*3' | GAGACTCCTGGAAAGATGCT |
| *dps*5' | CTGTTGATAACTTGCGTGGT |
| *dps*3' | GTTCATTGACCTGTCGCTG |
| *invA*5*'* | TGTCAGTCTCTTCCGTATGTG |
| *invA*3' | GCCTGCTCTTTGGTTTGT |
| *sipA*5' | AAAGATGGAAAGGTGGTCAC |
| *sipA*3' | CGGCTTCACATTCACAATC |
| *ompW*5' | CACTGACCTGACCCTCTTT |
| *ompW* 3' | CGCAATCAACTGTTCCAC |
| 16S5' | GGCAGGCTTGAGTCTTGTA |
| 16S3' | TGAGCGTCAGTCTTTGTCC |

**Supplementary Table 2**

**Table S2 The quality control of RNA sequencing of *S*. Typhimurium**

| Group | Samples | Raw Reads | Clean Reads | Error Rate (%) | Clean Q30 (%) |
| --- | --- | --- | --- | --- | --- |
| F-Non-O | F-Non-O-1 | 29633228 | 29344972 | 0.0248 | 94.63 |
|  | F-Non-O-2 | 23319258 | 22949014 | 0.0255 | 94.03 |
|  | F-Non-O-3 | 25836148 | 25554564 | 0.0251 | 94.41 |
|  | F-Non-O-4 | 27779934 | 27523600 | 0.0248 | 94.58 |
| F-AOS | F-AOS-1 | 28304466 | 27973202 | 0.0245 | 94.82 |
|  | F-AOS-2 | 26513374 | 26293132 | 0.0244 | 94.96 |
|  | F-AOS-3 | 26951914 | 26725808 | 0.0244 | 94.96 |
|  | F-AOS-4 | 26654660 | 26447846 | 0.0245 | 94.89 |
